# Supplementary material for: Prognostic Value of Neoadjuvant Chemotherapy in Patients with Borderline Resectable Pancreatic Carcinoma Followed by Pancreatectomy with Portal Vein Resection and Reconstruction with Venous Allograft
Source: J Clin Med. 2022 Dec 12;11(24):7380. doi: 10.3390/jcm11247380 (PMC9787949; doi:10.3390/jcm11247380)
Supplement: Supplementary file 1 [file jcm-11-07380-s001.zip › jcm-2055193-supplementary.pdf]

Supplementary material

Figure S1. Microscopic performances of vascular margin

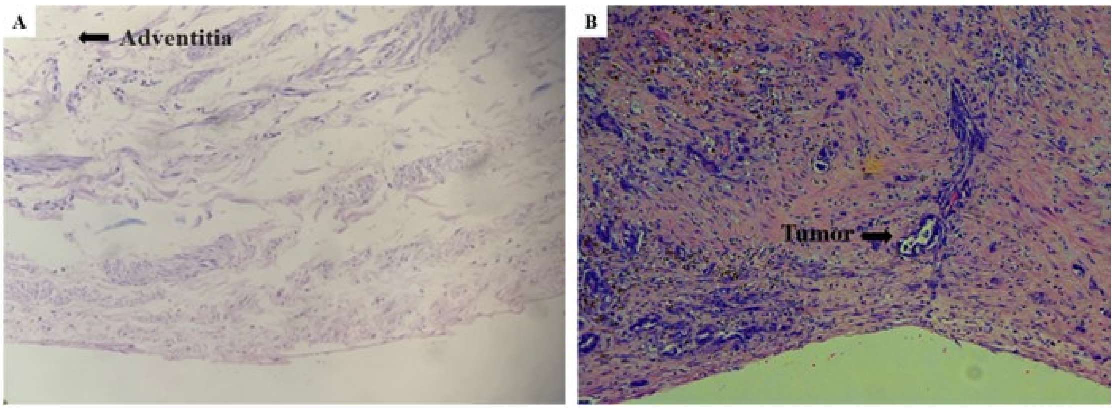

(A) Microscopic performance of vascular margin in Group1(HE staining ×100).
(B) Microscopic performance of vascular margin in Group2 (HE staining ×100). (Abbreviation: HE, hematoxylin and eosin)

Figure S2. CA19-9 level after operation with/without NAC.

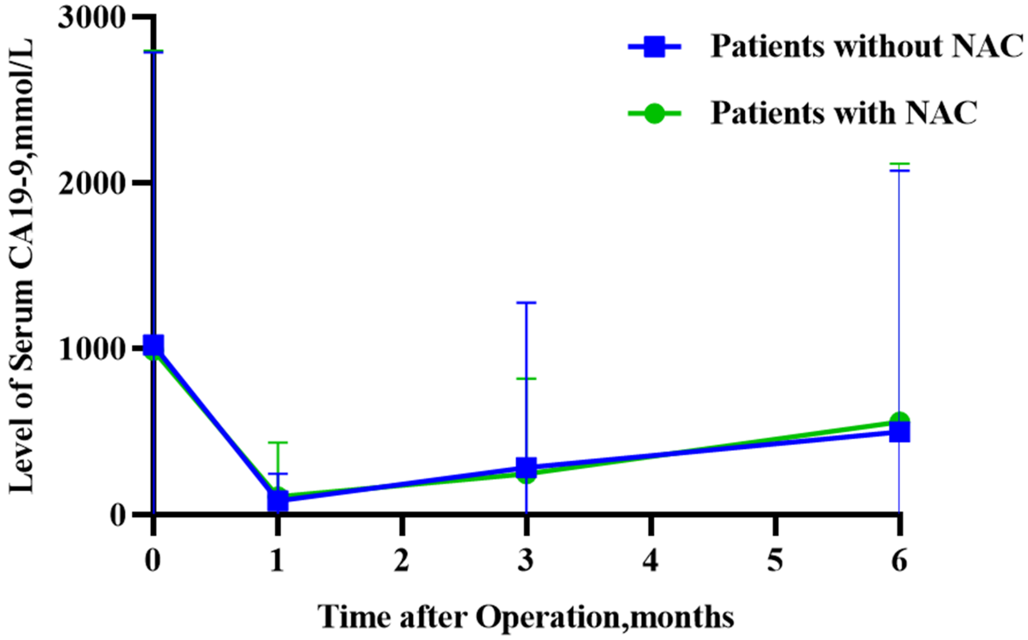

CA19-9 significantly decreased after operation, because of the tumor resection. However, CA19-9 increase gradually over time after operation.
